# *Bacillus anthracis* Factors for Phagosomal Escape

**DOI:** 10.3390/toxins4070536

**Published:** 2012-07-10

**Authors:** Fiorella Tonello, Irene Zornetta

**Affiliations:** 1 Institute of Neuroscience of the National Research Council, 35131 Padua, Italy; 2 Department of Biomedical Sciences, University of Padua, viale G. Colombo, 3, 35131 Padua, Italy; Email: irenezornetta@gmail.com

**Keywords:** *Bacillus anthracis*, anthrax, spore, germination, phagocytes, toxins, anthrolysin, phospholipase C, catalase, superoxide dismutase, capsule

## Abstract

The mechanism of phagosome escape by intracellular pathogens is an important step in the infectious cycle. During the establishment of anthrax, *Bacillus anthracis* undergoes a transient intracellular phase in which spores are engulfed by local phagocytes. Spores germinate inside phagosomes and grow to vegetative bacilli, which emerge from their resident intracellular compartments, replicate and eventually exit from the plasma membrane. During germination, *B. anthracis* secretes multiple factors that can help its resistance to the phagocytes. Here the possible role of *B. anthracis* toxins, phospholipases, antioxidant enzymes and capsules in the phagosomal escape and survival, is analyzed and compared with that of factors of other microbial pathogens involved in the same type of process.

## 1. Introduction

*Bacillus anthracis* is a gram-positive spore-forming bacterium that causes anthrax, an acute, often fatal infection [1,2]. *B. anthracis* is widely distributed on the earth’s surface in form of spores, hard-shelled, highly stable particles that can resist extreme conditions and be easily disseminated. Depending on the route of entry of the spores, human anthrax occurs as a cutaneous, inhalational or gastrointestinal infection. In any case, the *B. anthracis* spores are ingested by local phagocytes, which activate and recruit other immune cells. The spores germinate inside the phagosome and a battle begins between the host cell and the parasite. In most cases, the bacteria are destroyed, but sometimes, in some not well-defined circumstances, the invader prevails, leaves phagosome and destroys the host cell. If the phagosome activates a program of antigen presenting cells and migrates towards lymph nodes, it acts as a “Trojan horse” transporting the enemy inside the body fluids. From the lymphatic circulation, the bacterium reaches the blood, which is an ideal growth medium for *B. anthracis*. Here, it rapidly multiplies reaching 10^9^ organisms/mL blood and, in the absence of an immediate administration of antibiotics, death occurs from bacteremia and toxemia, as *B. anthracis* produces toxins that inhibit the innate and adaptive immune system [3] and a capsule that impairs phagocytosis [4].

In this review the weapons that *B. anthracis* uses to survive and multiply within the phagosome are analyzed and the circumstances in which this battle can be won by the parasite are discussed.

Anthrax has a very complex pathology, multiple factors are involved and many steps of the infection are not known. After penetrating into the respiratory or intestinal system or into a wound, the spores can germinate *in loco* or be phagocytized and germinate inside the phagosome. This aspect is still under discussion but the more diffused opinion is that, mainly in the case of inhalational anthrax, germination does not takes place at the spore penetration site but inside phagocytes, in alveolar macrophages or, with higher probability, outside the lungs in antigen presenting cells moving throughout the lymphatic system [5,6] as the lung are not an appropriate site for spore germination [6]. In contrast, in cutaneous and gastrointestinal anthrax, germination and growth of the bacteria happen at the initial site of spore entry [7,8] and at least in the case of cutaneous anthrax, in the extracellular space [7]. In this review only the case of germination inside phagosomes is considered and it is assumed that at the beginning of the infection, the anthrax toxic factors are released from inside the phagocytes and that these cells are not impaired by anthrax toxins from the outside. The recently proposed infectious routes via internalization and transcytosis of the spores in alveolar endothelium [7] or for disruption of the endothelial barrier have very recently been reviewed by Weiner and Glomski [8] and are not dealt with in this article.

## 2.*Bacillus Anthracis* Entry into the Phagocytes

Phagocytosis is started following interaction between transmembrane receptors on the extracellular membrane of the phagocyte and molecules on the surface of the *B. anthracis* spore. Binding and uptake of *B. anthracis* spores by phagocytic cells is a dynamic process—still not completely known—involving different receptors and multiple signaling pathways. CD14, an extracellular protein anchored into the membrane by a glycosylphosphatidylinositol tail, binds to rhamnose residues of BclA, a glycoprotein of the *B. anthracis* exosporium, and by involving TLR2 signaling, promotes inside-out activation of the integrin Mac-1 (CD11b/CD18) that somehow interacts with the BclA protein and functions as a co-receptor for the spore [9]. Accordingly, mice deleted for Mac-1 or for CD14 are more resistant to subcutaneous infection with *B. anthracis* spores [10]. Besides TLR2, other TLRs can be involved in *B. anthracis* spore phagocytosis: e.g., both TLR2^−/−^ and TLR4 deficient mice are reported to be resistant to aerosol exposure to *B. anthracis* spores [11]. Moreover BclA deleted spores are engulfed by macrophages to the same extent, and have the same virulence of wild-type spores [12] suggesting that other molecules in the *B. anthracis* exosporium should be recognized by phagocyte receptors. The main role of BclA can be to direct the spore towards the interaction with phagocytes, as spore mutants deleted for BclA present higher adherence to non-phagocytic cells than epithelial and endothelial cells and fibroblasts [13]. The interaction between *B. anthracis* spores and phagocytes also involves opsonins: IgG proteins have been found in non-immune human serum that bind anthrax spores and activate the classical pathway of complement activation, inducing the deposition of the opsonin C3b on the spore surface, a promoter of spore phagocytosis and survival in human macrophages [14]. Gu *et al.* very recently [15] found that the deposition of C3b on spore surfaces is not mediated by IgG in non-immune human serum, but is dependent on C1b recruited by the spore protein BclA. Moreover, complement fragment deposition and spore phagocytosis by mouse macrophages were significantly reduced in BclA lacking spores, and spores phagocytized via complement opsonization appeared to survive better than spores phagocytized by other mechanisms [15]. Other human serum proteins interact with *B. anthracis* spores, one of which, plasminogen, helps the evasion of the complement system [16]. Other studies will be necessary to understand the whole picture of the phagocytosis steps, to clarify these partially contradictory data, to understand if other receptors are involved and if the spores follow different destinies depending on the receptors involved in phagocytosis.

## 3. *Bacillus Anthracis* Factors that Can Interfere with the Phagosome Maturation Process

Following the internalization, the particle is localized within a nascent phagosome that undergoes different steps of maturation by fusions and fusion-fissions events, acquires a set of proteins and phospholipids, and becomes a complex structure able to destruct the pathogens [17,18]: the phagolysosome. This process of transformation is coordinated by proteins, such as the Rab GTPases, responsible for regulating the endocytic pathways, and four phases, similar to those characterizing the process undergone by endosomes, can be outlined: early, intermediate and late phagosomes and phagolysosomes. Inside the phagolysosomes acidic pH, exposure to digestive enzymes, high concentration of reactive oxygen species, nitrogen intermediates and antimicrobial peptides contribute to eliminate intracellular pathogens [19]. Pathogens escaping these digestive compartments can encounter other host defense mechanisms such as proteasome degradation and autophagy [20,21]. The inflammasome can also be activated with consequent modulation of the immune response and induction of host cell death as a defense mechanism [22].

Unfortunately, there are no studies of characterization of the phagosome containing the *B. anthracis* spore, except for co-localization studies, in RAW264.7 cells, with LAMP-1 [23,24] considered a marker of late phagosome and phagolysosomes [25]. The fact that a dominant-negative form of Rab7 blocks the fusion with lysosomes [26], and that inhibitors of the phagosome acidification, bafilomycin A and chloroquine, improve the survival of the pathogen [27], suggests that bacteria have to release factors that inhibit the phagosomal maturation to the final stage in order to survive, since once they have arrived in the lysosomes, they are destined to be digested. Transcriptional proﬁling of *B. anthracis* Sterne strain bacteria, isolated from within murine macrophages, highlighted the presence of 50 genes that are up-regulated during growth in host macrophages relative to growth *in vitro* [28]. Some of these genes are associated with metabolic adaptations to the intracellular environments (genes involved in iron acquisition and in biosynthesis of purines, some amino acids, biotin, NAD), others code for known virulence factors (anthrax binary toxins) or for protein homologs linked to the virulence of other microbial species. Examples of the last set include genes encoding a hemolysin, several phospholipases, adhesion lipoproteins and the catalase gene *katB*, as well as genes encoding several members of a multidrug resistance protein family. Other up-regulated genes are associated with the use of alternative electron acceptors indicating the the bacterium inside the macrophages is oxigen starved. All these data indicate that *B. anthracis,* inside the macrophages, triggers a specific response very relevant to anthrax pathogenesis.

**Figure 1:**
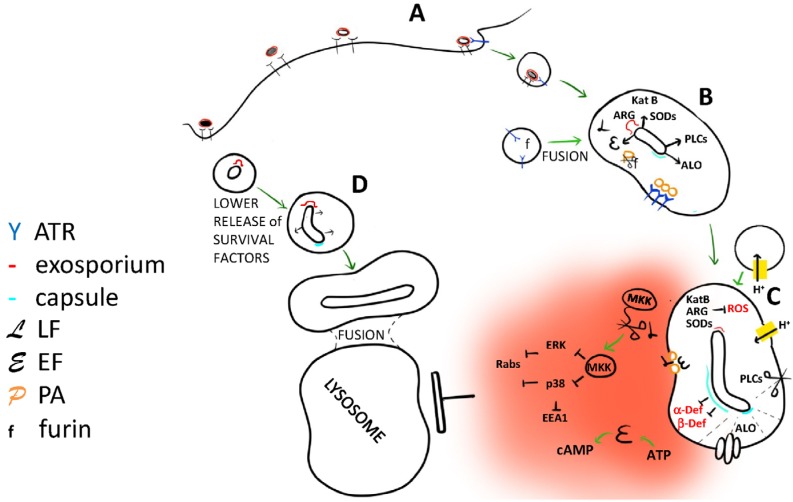
Factors produced by *B. anthracis* having a possible role in helping bacterial survival inside the phagosome. (**A**) *B. anthracis* spore is internalized after interaction with membrane receptors, Mac-1 or other receptors (see introduction). It can also interact with anthrax toxin receptors (ATRs) as protective antigen (PA) can be present in the exosporium; (**B**) Once inside the phagosome, the spore, in response to intracellular factors, is stimulated to germinate and activate a program of protein production different from that activated in *in vitro* culture; (**C**) The microorganism releases factors that prevent the maturation of the phagosome and help the bacterium to leave it. LF and EF inhibit the fusion of the phagosome with the lysosome (see text). This fusion can also be inhibited by the polyglutamic acid of the capsule that moreover inhibits the action of antibacterial alpha- and beta-defensins. The increase of oxidant species (ROS) into the phagosome is inhibited by different factors produced by *B. anthracis*: an arginase and two superoxide-dismutase SODs proteins present into the exosporium, and a catalase (KatB) abundantly produced during the germination phase inside the phagosome. Finally the poly-γ-D-glutamic acid of the capsule, the cytolysin ALO and three PLC proteins can contribute to the destabilization and lysis of the phagosomal membrane and so to the exiting of the bacterium into the cytosol; (**D**) The *B. anthracis* phagocytized in the germination phase (grey and black spores) probably activates a different germination program with insufficient production of survival factors.

Among the genes that are up regulated by *B. anthracis* inside the phagocytes, we describe the ones that can be involved in the mechanism of survival of the pathogen inside the phagosome (see Figure 1).

## 4. Lethal and Edema Toxins

The vegetative form of *B. anthracis* secretes three polypeptides, called protective antigen (PA), lethal factor (LF) and edema factor (EF), that combine to form the two well-known exotoxins, lethal toxin (LeTx: PA + LF) and edema toxin (EdTx: PA + EF). PA, after the removal of a 20 kDa fragment on its *N*-terminal side by furin-like enzymes, binds to one of its cell-surface receptors, anthrax toxin receptor 1 (ATR1) or capillary morphogenesis gene 2 (*CMG2*), and forms a homo-oligomer that interacts with EF and LF [29]. The internalization of this complex into endosomes is followed, when the pH is acidified by the proton pump, by the insertion of PA oligomer into the endosomal membrane and the translocation of EF and LF through the PA pore into the cell cytosol [30]. EF, a calmodulin-dependent adenylate cyclase, causes a prolonged increase of cyclic adenosine monophosphate (cAMP) concentration into the cytosol [30]; LF is a metalloprotease which cleaves and inactivates most isoforms of mitogen-activated protein kinase kinases (MAPKKs, also known as MEKs) blocking the kinase signaling cascade and therefore the activation of the mitogen-activated protein kinase (MAPK) p38, ERK and JNK [31,32].

Anthrax binary toxins have multiple effects on phagocytic cells e.g., they inhibit the phagocytic ability of macrophages [33] and impair the maturation and chemotaxis of different phagocytes and their inflammatory cytokine secretion [3,34,35]. LeTx causes necrosis of macrophages isolated from some mouse strains, depending on the alleles of the inflammasome sensor Nlrp1b, but contrary to all expectations, mice that have macrophages more sensitive to LeTx are more resistant to *B. anthracis* [36,37,38]. It has been suggested that the death of the phagocytic cells is not a mechanism of virulence of the pathogen but an immune-response of the host, mediated by the inflammasome, to stimulate a larger inflammatory cytokine release and the consequent recruitment of more immune cells, in particular of neutrophils [37,38] which are known to have an important role in controlling *B. anthracis* infection [39,40]. Reinforcing this idea, very recently, it has been found that at least another gene of murine chromosome 11 is involved in the control of the severity of the host response to LeTx and to other inflammatory stimuli [41].

Lethal and edema toxins can also have a fundamental role in the bacterial survival within phagocytic cells. The anthrax bacilli expresses the toxin genes rapidly after germination [42,23], and these are among the genes up-regulated by *B. anthracis* inside macrophages in the *in vitro* expression [28]. 

The anthrax toxin receptor *CMG2* is ubiquitous and could also be present inside the phagosome containing the spore [43,44]. Moreover PA receptors can be involved in the internalization of the spore, as PA is present as contaminant in the exosporium [42]. The PA oligomer formation requires the activation of PA by the cleavage of its *N*-terminal 20 kDa side, a cleavage that is performed by furin or furin like enzymes in the extracellular space or in the serum [45,46]. How can PA be activated inside the phagosome? There is evidence that furin is localized in multiple protein-processing compartments, principally in the TGN, and that it cycles among sorting compartments, the cell surface and the early endosomes. Furin has a broad pH optimum (more than 50% of enzymatic activity between pH 5 and 8) and, in mildly acidic early endosomes, is known to cleave different substrates including diphtheria toxins, shiga toxin and *Pseudomonas* exotoxin A [47,48]. Therefore, when the internalized spore geminates and the bacteria releases the binary toxins, PA can be activated by furin inside the phagosome, interact with *CMG2*, form the multimer (Figure 1B) and, when the lumen of the organelle is acid enough, insert into the membrane and translocate LF and EF into the cytosol (Figure 1C).

Both LF and EF activities are able to interfere with the signaling involved in phagosome transformation: The fusion events necessary for the maturation of this organelle to the phagolysosomal phase involve an actin polymerization process that, at least in the case of *Mycobacteria,* was shown to require p38 kinase activity [49] and to be inhibited by cAMP increase [50]. Different effectors of the endocytic pathways, as for example EEA1 and Rab5, are regulated through phosphorylation by p38. Moreover, p38 and ERK induce the up-regulation of the expression of Rab proteins that are known to be involved in the endocytic pathways [25]. 

Furthermore, anthrax toxins inhibit the expression in macrophages of type-IIA secreted phospholipase A2 (sPLA2-IIA), one of the major components involved in innate host defense against bacteria [51,52], especially against gram positive bacteria. It is known that manipulation of host lipid metabolism alters phagosome maturation and promotes bacterial survival within these organelles [53]. 

A direct observation of the toxins exiting from the anthrax spore containing phagosome has never been reported, but spores in RAW264.7 cells co-localize with ATRs-EGFP. Moreover, RAW264.7 cells deleted for *CMG2*, the only ATR expressed in that cell line [24], are resistant to infection with *B. anthracis* spores; and RAW264.7 cells deleted for the ATRs were able to control a challenge with *B. anthracis* in mice, if inoculated in the peritoneum before the spores [54].

The *B. anthracis* spores co-localize with LAMP-1 both in wild type and in RAW264.7 cells deleted for *CMG2*, suggesting that the binary toxins do not alter the maturation of the phagosome, at least until the late paghosomal phase, where the proton pump is also acquired and acidification begins [24].

Finally, anthrax toxin expression and release into the cytosol of phagocytic cells was shown to alter the process of autophagocytosis, but the effects reported for LF and for EF are conflicting, enhancing for LF [55,56] and inhibiting for EF [57]. Further studies will be necessary to define the combined effects.

## 5. Anthrolysin O

In addition to LeTx and EdTx, *B. anthracis* secretes a toxin called anthrolysin O (ALO) that is a hemolysin and a cholesterol-dependent cytolysin (CDC). CDCs are pore-forming toxins, which require cholesterol in the membrane to form pores with a mechanism not completely clarified. It is generally known that monomers oligomerize into a prepore complex and that this step is followed by a large conformational change in each oligomer, resulting in the insertion into the membrane [58]. 

ALO is hemolytic [59], has lytic activity against phagocytes [60,61] and decreases the barrier function of human polarized epithelial cells [62,63]. Its expression in culture supernatants is highly influenced by growth media and this suggests that expression and/or secretion of ALO may be under environmental control [59]. Both transcriptional proﬁling [28] and RT-PCR expression experiments of newly germinated bacteria after phagocytosis of the spores by mouse macrophages [64] demonstrate that the ALO expression pattern is similar to that of the PA gene, and is controlled by anaerobic conditions. 

CDCs appear to play a significant role in the pathogenesis of a variety of gram-positive species, including *Bacillus cereus*, *Listeria monocytogenes*, *Clostridium perfrigens*, *Streptococcus pneumoniae* and *Streptococcus intermedius* [65]. Listeriolysin, a CDC of *Listeria monocytogenes* is responsible for the escape of bacteria from the phagosome to colonize the cytoplasm [66]. ALO is able to complement the escape phenotype in a *Listeria monocytogenes* strain lacking LLO [67] and although deletion of ALO alone in *B. anthracis* yielded no differences in virulence in mice, the deletion of ALO gene together with those of the three phospholipases described in the next paragraph resulted in a clear attenuation of bacterial virulence, growth and survival in the phagocytes [68]. These data indicate that ALO can be one of the tools used by *B. anthracis* to destabilize and break the phagosomal membrane.

## 6. Phospholipases C

Three genes were annotated on the *B. anthracis* genome that encode three putative phospholipases C (PLCs), a phosphatidylcholine-specific (PC-PLC), a sphingomyelinase (SMase), and phosphatidylinositol-specific PLC (PI-PLC) [69]. As for other virulence factors, *B. anthracis* PLCs are induced by strictly anaerobic conditions and are expressed at the early stages of infection within macrophages [64]. The disruption of all three PLC genes is necessary to obtain symptom attenuation in a murine model of anthrax and reduction of the bacilli growth and survival in macrophages [70]. As reported in the previous paragraph, a more efficient attenuation in cell culture and in mice challenged with *B. anthracis* spores was obtained by deleting both ALO and the three PLC genes, meaning that these proteins have overlapping and synergic roles in the anthrax pathogenesis [68]. A cooperation and synergy of these proteins was also found in hemolytic tests on human RBC [64] and it is known that in *Listeria monocytogenes* PLCs are used in combination with the pore-forming cytolysin to disrupt phagosomal membranes and aid the escape of the bacterium into the cytosol [71]. This functional redundancy and synergy between PLCs and CDCs implies that their activities are relevant for the pathogenesis of these intracellular or partially intracellular bacteria.

## 7. Antioxidant Enzymes

Following phagocytosis of microbes, macrophages use oxidants as part of their microbicidal activity. It is noteworthy that they up-regulate nitric oxide synthase (NOS 2), the enzyme that metabolizes L-arginine to L-citrulline and nitric oxide (NO); moreover, NOS 2 reduces O_2_ to the superoxide ion (O_2_^•−^) and H_2_O_2,_ ·NO and O_2_^•−^react to generate peroxynitrite (ONOO–). The reactive oxygen species (ROS), especially ·NO, contribute to the digestion and elimination of *B. anthracis* by macrophages [72], however both *B. anthracis* endospore and vegetative forms possess various tools to contrast these reactions.

*B. anthracis* exosporium contains an arginase, an enzymes that metabolizes L-arginine to L-ornitine and urea, which decreases NO radicals produced in macrophages [73,74]. The closest homolog of the *B. anthracis* arginase is that from *Helicobacter pylori* which inhibits host nitric oxide production, allowing for survival of the organism when co-cultured with activated macrophages. However, the *B. anthracis* arginase gene did not complement a *Helicobacter pylori* arginase mutant: characteristic differences in arginases of these two bacteria may reflect distinct *in vivo* niches occupied by the organisms [75].

The *B. anthracis* genome encodes four different SODs, enzymes that catalyze the dismutation of O_2_^•−^ to hydrogen peroxide and molecular oxygen. SODs belong to different structural classes based on metal cofactor specificity and have distinct functions to protect the bacterium from exogenously and endogenously produced oxygen radicals, depending on their localization in the bacterial periplasm or cytoplasm, respectively. SODs of each class contribute to the virulence of many pathogens including *Salmonella enterica*, *Serovar typhimurium*, *Mycobacterium tuberculosis*, *Staphylococcus aureus*, *Streptococcus agalactiae*, *Francisella tularensis*, *Neisseria meningitidis*, *Brucella abortus*, and *Enterococcus faecalis*. The four *B. anthracis* SODs belong to three different structural classes and two of them are localized in the exosporium. They are functionally redundant, and signiﬁcant attenuation (40-fold) of bacterial virulence upon intranasal challenge of mice was seen only with the deletion of all four SODs genes [76].

One of the first and most expressed genes by *B. anthracis* spores during the germination within macrophages is *katB*, a catalase gene having homologs in other species and involved in protecting the bacteria from host-derived reactive oxygen intermediates [28,77]. *B. anthracis* catalase, an enzyme that converts hydrogen peroxide to water and oxygen, is activated by the production of NO by the bacterial NOS (bNOS) which accumulates in the spore during the sporulation phase [28]. In addition to catalase activation, NO interrupts the production of damaging hydroxyl radicals from the Fenton reaction, a process responsible for generating hydroxyl radicals that react with DNA bases, sugar moieties, and amino acid side chains, causing various types of lesions. Anthrax spores deficient in bNOS lose their virulence in a mouse model of systemic infection and exhibit severely compromised survival when germinating within J774 macrophages [78].

The ability of *B. anthracis* to subvert ·NO production has important implications in the control of infection, and further studies are warranted to evaluate the role of the antioxidant enzymes in anthrax pathogenesis.

## 8. The Capsule

*B. anthracis* produces a plasmid-encoded anti-phagocytic poly-γ-D-glutamic acid capsule, which is essential for virulence, surrounds the vegetative bacterium form and is a primary mechanism of immune cell evasion. Although the mechanism by which the capsule inhibits phagocytosis is not well established, other bacterial capsules are known to inhibit phagocytosis by their anionic charge and by shielding potential bacterial surface adhesins [4]. The capsule of *B. anthracis* is produced as high-molecular weight form (>100 kDa), which is first polymerized on the bacterial cell surface *in* *vivo* and subsequently degraded to the lower-molecular weight capsule (<14 kDa). The latter is released from the bacterial cell surface into the culture supernatant and it has been proposed that this process may be an essential mechanism acting as a decoy against host defense systems, especially complement activation [79].

In addition to its antiphagocytic properties, the capsule may contribute to resistance of bacilli to phagocytes in evading host innate immunity. It can block bactericidal activities of neutrophil extracts and of some cationic peptides, including α- and β-defensins. The treatment of encapsulated bacilli with a capsule de-polymerase facilitates their efficient killing by neutrophils both *in vitro* and *in vivo* [80,81]. In mycobacterium, the polyglutamic acid that composes the capsule is implicated in the phenomenon of fusion inhibition between phagosomes and lysosomes [82,83] and it possesses membrane-destabilizing properties that allows biomacromolecules endosomal escape, similarly to other poly-anions employed in drug delivery [84].

In an anthrax infection, the kinetics of capsule production during germination of *B. anthracis* inside phagocytes is not known and the transcriptional profile of *B. anthracis* bacteria isolated from within murine macrophages, described in the first paragraph of this review [28], was performed on the Sterne strain that is a non-encapsulated natural variant. When *B. anthracis* cells are grown *in vitro* the capsule is highly expressed in an elevated CO_2_/bicarbonate (5%) concentration, a condition similar to the phagosomal inner micro-ambient. Moreover, AtxA, the transcriptional regulator of the synthesis of the three toxin components, also controls the synthesis of the surface elements, capsule and S-layer. Recently, the capsule antigenic level, over the course of inhalational anthrax in rhesus macaques, was shown to follow a triphasic kinetics, a kinetics similar to that of the lethal factor [85].

## 9. In the Battle between Phagosome and *B. Anthracis*, What Circumstances Facilitate the Pathogen?

**Figure 2:**
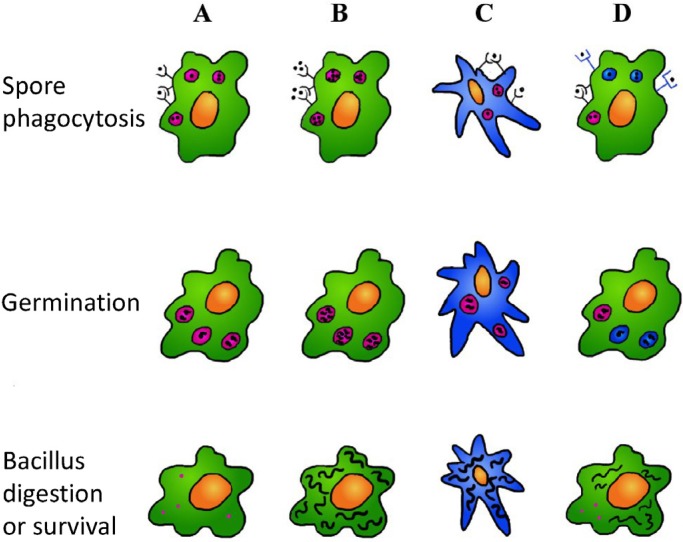
Conditions that can favor the survival of *B. anthracis* in the phagosome. (**A**) The *B. anthracis* microbes internalized as spores by phagocytes are, in most cases, digested after spore germination. However in some, still not completely understood, circumstances the bacterium can survive the phagosome digestion, leave the phagocyte and give rise to the dangerous bacteremia and toxemia typical of acute anthrax. These conditions can include; (**B**) high spore charges; (**C**) internalization by dendritic cells that, coherently with their action of antigen presenting cells, activate a milder digestion process; (**D**) internalization by receptors that are involved in a different phagosome maturation process.

Various experiments have shown that macrophages are very efficient in the elimination of *B. anthracis* spores (Figure 2A) [26,86] in particular the germinated ones [87,88], moreover, alveolar macrophage-depleted mice are more susceptible to *B. anthracis* infection [86]. These observations are clearly in contradiction with the idea that macrophages act as “Trojan horses”, transporting the pathogen inside the system. Neutrophils also are efficient killers of *B. anthracis* [38,39]. Cote and collaborators [6,89] suggest that neutrophils have a secondary, but necessary, role in the activation of a fully functional innate immune response to spores; others [39,40] observed that the high recruitment of neutrophils in anthrax skin infections can explain why this infection often resolves spontaneously while in inhalation anthrax, where there is little neutrophil infiltration, the infection rapidly develops and leads to sepsis and death.

Then, if phagocytes are generally so efficient in eliminating the ingested bacteria, it is obvious to ask when and why *B. anthracis* is able to breach the phagocyte from within. It has been observed that macrophages loaded with a low spore number have a high probability to be sporicidal [54,60], so a condition influencing the phagocyte infection outcome can be the spore burden (Figure 2B). Another possible explanation is that dendritic cells are the weak point (Figure 2C). Immature human dendritic cells are able to internalize non-germinated *B. anthracis* spores by means of coiling phagocytosis and studies on mice intranasal or intratracheally infected with *B. anthracis* spores have shown that lung dendritic cells transport spores to the thoracic lymph nodes [90,91]. In a comparison of the bactericidal activity of primary enriched bone marrow dendritic cells or primary bone marrow macrophages infected with *B. anthracis* spores and cultured dendritic cells showed a poor ability to eliminate the pathogen and to provide an environment capable of allowing bacterial growth [91]. The efficiency of dendritic cells in bacterial killing could be lower than that of macrophages in light of the fact that they must have milder digestion conditions. Dendritic cells have to present proteolytic peptides derived from pathogens to T cells and so they activate, in respect to other phagocytes, a tight control of phagosome content processing, a near-neutral pH environment, with less lysosomal proteolysis and lower production of reactive oxygen species [92,93]. All these features contribute to creating an internal environment more appropriate for pathogen survival compared to the phagosome of macrophages and neutrophils. This can also explain why inhalational anthrax has such a deceptive pathology, characterized by feeble local symptoms, like those of a common flu, that then rapidly evolve into a dangerous bacteremia and toxemia [1,2,94]: Macrophages (and/or neutrophils) can control the local infection but at the same time dendritic cells, acting as ‘Trojan horses’, can transport the enemy inside the host. Moreover, the lung dendritic cells just have to cross a thin layer of epithelial or endothelial cells (about 200 µm) to reach the circulatory system where bacteria find their favorite medium to grow and where a higher CO_2_ concentration promotes an expression increase of many bacterial factors, capsule and binary toxins included.

A last possibility is that the spores are internalized by interaction with different receptors and that, depending on the receptors involved, they undergo a different fate (Figure 2D). For example *B. anthracis* can only block the phagosome maturation if there are enough PA receptors in the phagosomal membrane and these can only be present if involved in an interaction with PA in the spore exosporium; or it can be that only with some internalization pathways, the nascent phagosome is fused with vesicles containing PA receptor and/or furin-like enzymes (Figure 1A,B). Other receptors that make the phagocyte more vulnerable are CD14 and Mac-1: Macrophages deleted for these receptors are more resistant to spore infection [10]. Spores phagocytized via complement opsonisation, and then via Mac-1/CR3 receptor, appeared to survive better than spores phagocytized in presence of heat inactivated serum [15]. In contrast, when the spores are internalized as a consequence of opsonisation with PA antibodies, the macrophages have a higher chance to digest the pathogen [42]. Plasminogen deposition, described by Chung *et al.* [16], can be adopted by the spore to avoid phagocytosis, as interpreted by the authors, but the real aim can be that of favoring the phagocytosis by a way that allows pathogen development. In fact, the interaction with different receptors can activate macrophages in different ways with the consequent release of different cytokines that have a decisive role on the modulation of phagosome biogenesis and activity [25].

The involvement of different receptors can explain why germinated spores are more easily eliminated by the macrophage compared to ungerminated ones: It could be that, following the germination, other molecules are exposed on the pathogen surface that cover, or preponderate over, the exosporium molecules, allowing a different internalization pathway. But the motif of the higher sensitivity of macrophages to the ungerminated spores can also be the environment in which the spores begin their germination: The program of gene expression triggered when the spore germinates inside the phagosome includes an up-regulation of genes useful for pathogenic offence that, when the spore germinates outside the cell, can be expressed at too low doses, not sufficient to stop the digestion process of the phagocytes (Figure 1).

## 10. Conclusions

Notwithstanding the idea that *B. anthracis* is transported towards lymph nodes by macrophages that has been proposed for a long time [5], the mechanisms adopted by the vegetative form of the anthrax bacterium to resist the digestive process of the phagosome and leave it are still unknown. Here different *B. anthracis* factors are described that, according to their activity, or by comparison with factors of other pathogen, can contribute to this process (Figure 1). Lethal and edema toxin together with the poly-γ-D-glutamic acid of the capsule can inhibit the fusion between phagosomes and lysosomes; the poly-γ-D-glutamic acid capsule inhibits the attack of alpha and β-defensins while exosporium arginase and four superoxide dismutases released by the vegetative bacterium can keep the oxidative burst activation under control. Finally, three phospholipases C, the capsule poly-γ-D-glutamic acid and the pore forming toxin ALO can help the disassembly of the organelle membrane to create a sorting passage for the bacillus.

To date, the phagosomes containing *B. anthracis* spores or bacteria have not been characterized for their protein and lipids content to understand if and how the bacterial released factors alter their properties. The study of the composition of pathogen-containing phagosomes is complicated, as the pathogens cause an alteration of their hosting organelle, giving them a mixed identity. Many markers should be used, as a partial, simplified characterization can be misleading. A global approach, such as phagosome purification and proteomics, should be adopted, while immunoﬂuorescence can provide only partial information [25].

Even the knowledge of the various cell receptors and exosporium molecules involved in spore/phagocyte interaction and internalization has to be expanded to know if the infection outcome is different depending on the involved surface proteins. Finally, it will be relevant to know the composition and activity differences of the phagosomes in the various phagocytes.

All this information will allow a better understanding of the mechanisms adopted by pathogens to overcome the phagocytic attack and will help in the attempt to find novel strategies to stop their infection.
